# Effort Syndrome

**Published:** 1918-12

**Authors:** Alfred E. Cohn


**The Effort Syndrome.** By Lt.-Col. Alfred E. Cohn, M. C.

It is difficult to define the Effort Syndrome. The term has been adopted to avoid assuming that its exact etiology, its exact symptomatology, or its exact therapy, are understood. A great deal is known about the manifestations of the affection, but it requires further analysis and study.

The symptoms which it is now proposed to include in this conception have at one time or another been known as the Irritable Heart of Soldiers, the Tropical Heart of Soldiers, and Disordered Action of the Heart (D. A. H.). Because of the vagueness of the new term, suggested by Thomas Lewis, its introduction requires explanation. It has been the uniform observation of physicians concerned with the care of cases in this group that recovery is distinctly retarded if the diagnosis directs attention to the existence of a defect in any organ or system of the body. Patients to whom it is suggested that a cardiac affection may be present rivet their attention on the heart so firmly as to render it difficult and sometimes impossible to provide for their recovery. Directing attention to derangement of the nervous system is accompanied by like untoward consequences. From the point of view of the cardio-vascular system, discarding the term D. A. H. has further advantages. It serves the interests of clearer medical thinking. For no one has yet suggested that a structural change takes place either in the myocardium or in the endocardium.

But the complaints of patients have, since the condition was first recognized, focussed attention on the heart. A sense of fatigue, shortness of breath, pain in the chest, palpitation, and nervousness are the most common reasons for reporting at sick call. A number of other complaints are sometimes added to these; sleeplessness, bad dreams, headache and shakiness, attacks of fainting, and sometimes fainting with unconsciousness. The complaints are often out of proportion to the manifest robust appearance of the men. Some Individuals may complain of a single symptom, others of more. The physical signs vary in some respects with the severity of the affection. The fascies suggests the appearance commonly associated with an anxiety neurosis, — anxious eyes, drawn mouth, puckered forehead. Shakiness of the hands, sometimes of arms and legs, — rarely of head and torso, — attract attention. Pharyngeal and corneal reflexes are diminished; the patellar reflex is exaggerated. Areas of skin hyperesthesia and hypoesthesia are present — for the most part irregularly distributed. Skin irritability is present, as is shown by the tendency to dermatographia. The examination of the cardio-vascular system is striking both for the positive findings and for what one fails to find. The symptoms naturally direct attention to this aspect of the examination. Symptoms referable to this system are indeed so prominent that the explanation for them has almost uniformly been sought in an abnormal function of the heart, or in an alteration of its structure. As a matter of fact, the heart is not larger than one finds it to be commonly in soldiers. Soldiers in any case have larger hearts than civilian controls. At the apex there is often a purr or small thrill, but this, although it has been supposed to precede the apex impulse, appears to be systolic in time. It is important to decide that this is so, and that it is not synchronous with auricular systole. So far satisfactory evidence on this point is wanting. To correspond to this curious thrill, one hears an impurity of the first sound — a splintering into serrations of its muscular portions. The first sound is often loud and thumping. The two phenomena give the impression of the sound and murmur of mitral stenosis. The second sound is usually not accentuated. If the subject is asked to exercise, the impurity of the first sound usually disappears and the second sound does not become accentuated. This test does much to remove the suspicion that the diagnosis of mitral stenosis need be entertained. The pulse rate in 75 o/o of the cases is elevated above 90; on the other hand, attention should be called to the fact that in 11 o/o it is below 80. But the rate is usually not maintained at a constant level — in most cases it fluctuates, and in some it does so violently and frequently. The blood pressure, both systolic and diastolic, is usually not elevated. Soon after the inception of a more rapid rate, one expects to see the pressure rise; that is a uniform experience, in the laboratory and in the clinic, in the study of cases of paroxysms of tachycardia, for instance.

There are two diseases, especially, from which the Effort Syndrome must be distinguished — mitral stenosis and hyperthyroidism. The distinction is in neither instance simple. In the latter stages there is usually little difficulty in making a satisfactory diagnosis. But in the early stages, difficulty is experienced. Sir James Mackenzie has long been in the habit of pointing out that progress in clinical medicine often rests on the development of knowledge of *symptoms* in the incipient stages of diseases. Here especially the difficulty in diagnosis is experienced in the early stage. Even small straws pointing the way to correct interpretation are important. An exercise test has been of value. Exercise, for instance, usually fails to accentuate the physical signs anticipated if mitral stenosis is present — the thrill, the murmur, and the accentuated second sound. On the contratry, in the Effort Syndrome these signs tend to diminish in intensity. Unfortunately neither the clinical history nor exact mechanical devices have been useful in facilitating a decision.

To distinguish the Effort Syndrome from hyperthyroidism is more difficult still. Stress must be laid on the diagnosis by means of the early symptoms. I recall the cardinal symptoms of hyperthyroidism; exophthalmus, thyroid tumor, tremor, tachycardia, diarrhea, sweating. There can be no doubt that the Effort Syndrome cases present manifestations strikingly like these, except that genuine exophthalmus is rare. Unquestionably many cases have enlarged thyroid glands, but a greater number have not. The tremor in the Effort Syndrome cases is not fine as in Graves disease, but is a shake, more or less violent. Tachycardia is absent in 10 o/o of the cases. Diarrhea is not common. In short, the signs are similar, but they are not identical. A striking difference between the Effort Syndrome and hyperthyroid cases is seen when the cases are at rest or asleep, and are free from effort. When tachycardia associated with breathlessness is present in hyperthyroidism, these persist at such times. But in the Effort Syndrome, especially when this is uncomplicated by gas poisoning, these phenomena disappear; the patient sleeps flat without discomfort: his pulse rate drops to normal. It is too much to say that these criteria are decisive. That a real difficulty is present was appreciated when the work at U. S. General Hospital N° 9 at Lakewood was planned. One phase of the study there was designed to throw light precisely on this subject. Major Peabody has been supervising this work. I am not now prepared to give you his results.

We are then in position to say that in the Effort Syndrome we do not deal with organic heart disease. As to its relation with hyperthyroidism we entertain a mental reservation. And in this respect we appreciate the fact that a physiological process may exist not as a thing in itself, but in response to a stimulus which sets a chain of events in motion. Under these circumstances it becomes necessary to inquire under what circumstances the Effort Syndrome arises, and with what other affections it is associated.

Individuals suffering from widely different causes complain of fatigue, pain in the chest, dyspnea, palpitation. Every one has known such complaints in convalescence after *acute infectious diseases* — typhoid fever, dysentery, pneumonia, trench fever, rheumatism.

Next come cases of *gas poisoning*. These are especially liable to this complex, whether the injury has been slight or severe.

The form of affection, from time to time, called *heart strain* should not be overlooked. The soldier under these circumstances suffers from the strain to which he is subjected. There is involved no disease; no neurosis; it is a simple mechanical defect from which he suffers. And finally there is a large group which may be designated as *Constitutional Effort Syndrome* cases.

There has been a tendency to associate the occurrence of the Effort Syndrome now with one, now with another disease as its causative agent. The theories that it was rheumatism, trench fever, or other special infection have each had adherents. There has been an acidosis theory as well.

But let me point out that early in the war it was evident to good clinical observers that what we now call the Effort Syndrome was not a new thing, but the exaggeration of an old one. Sir James Mackenzie has described its main features as has Thomas Lewis. The complex, as a matter of fact, presents in some respects the features of anxiety neurosis. To fasten to it, therefore, a term involving disorder of specific organs, as is done in the term D.A.H., or a term involving a definite physiological conception, as neurocirculatory asthenia, is unfortunate. It is probably better for the moment to consider the complex neither as an affection of the heart, nor as an infection, nor as a complex having a definite physiological basis, in the usual sense. To make this decision has important consequences, both in prognosis and in treatment.

If we were to look for the root of the difficulty in the war itself, we should perhaps come to understand certain phases of the affection better. The war furnishes abundant stimuli in the train of which follow phenomena such as have been described. They resemble closely those understood to represent anxiety states in civil life. The most common reaction to this stimulus has always been cardio-vascular-flushing, palpitation, a sense of fatigue (we call it weakness in the knees), a catching of the breath, and sometimes cardiac pain. But the cardio-vascular is not the only reaction with which we are familiar. The gastro-intestinal reaction is not uncommon — in women commoner than in men — but it has been encountered with moderate frequency in soldiers here in France. Still another reaction is musculo-skeletal in which contractures occur. The careful taking of histories will often show that the nature of the reflex to be anticipated under stress might have been predicted — less accurately in the cardio-vascular cases, because that is apparently the usual response and therefore less easy to be certain of, but strikingly in the gastro-intestinal and musculo-skeletal types.

Why so great a number of cases of gas poisoning develop the Effort Syndrome is difficult to explain. The fact may be pointed out that surgical casualties present this complex relatively infrequently. But in gas poisoning the agent is invisible and impalpable. The degree of injury is often unequal in severity in a group equally exposed. Curiously enough the Effort Syndrome develops most frequently in hospital during convalescence. The nature of the injury and leisure to reflect upon it appear to serve as the basis for the development of the symptoms. It is of course understood that certain chest injuries and burns appear after gas poisoning, and that these cause symptoms. But the Effort Syndrome is developed over and above these.

If the thought is correct that in the Effort Syndrome one is not dealing with an organic disease, nor with a physiological defect in the common sense, then this conception has an important bearing on the management of the affection and on the outlook for recovery.

The gassed cases present a separate and important problem. It does not appear to us quite clear what future, immediate or remote, may be predicted for gassed cases after they have been discharged from hospital as " A " men. It has been difficult to obtain accurate information of their subsequent behavior.

The management of the Effort Syndrome itself presents another and more difficult problem. It has been the intention to convey the idea that there is involved in these patients both a physical and a psychic factor. Which of these is fundamental and predominant it is difficult to decide. That arrangements must be at hand to provide for physical rehabilitation requires no discussion. That is universally admitted. But duration of treatment in hospital is subject to discussion. Effort Syndrome cases, in which it is clear no abnormal physical signs are present, must not be kept in hospital. In its atmosphere they do not improve, they deteriorate in morale. The proper place for their management is the Convalescent Camp. The gas cases with Effort Syndrome symptoms we believe should be transferred to the Convalescent Camps for the same reason, as soon as it is clear that no danger from respiratory infection is present. There should naturally be no fever nor any unhealed burns. In the interest of precaution the transfer should not be made until 10 days after the gas attack. Especial attention appears, however, to be required in the re-establishment of psychic integrity. All that can be put into convalescent camps to make for cheerfulness, to suggest comfort, to lead to a sense of duty in citizenship, to exhort and to encourage, should find place there. These ends cannot be accomplished by physical arrangements alone; they must be supplied by permeating the atmosphere of the camp with the things of the spirit. In so far as the Camps succeed in supplying that stimulus, genuine achievements in therapeutics may be anticipated.

The Effort Syndrome, then, is not a clearly defined clinical entity. Its manifestations are not simple, but embrace complaints referable to more than one organ and more than one system in the body. On this account it is necessary always to guard against making diagnoses of organic diseases having similar symptoms. The distinctions are often puzzling, for the complaints are often vague. One must remember that psychic disturbances are present and may possibly form the essential underlying factor of the complaint. No system of therapeutics can be considered adequate which does not take all these facts into account. We mean, in the convalescent camps now coming into being, to introduce whatever aids are available, in the attempt to treat this condition physically and psychically.

